# The effects of alpha-glycerylphosphorylcholine, caffeine or placebo on markers of mood, cognitive function, power, speed, and agility

**DOI:** 10.1186/1550-2783-12-S1-P41

**Published:** 2015-09-21

**Authors:** Adam G Parker, Allyn Byars, Martin Purpura, Ralf Jäger

**Affiliations:** 1 Department of Kinesiology, Angelo State University, San Angelo, TX, 76909, USA; 2 Increnovo LLC, 2138 E. Lafayette Pl., Milwaukee, WI 53202, USA

## Background

Alpha-glycerylphosphorylcholine (Alpha-GPC) and caffeine supplementation have been shown to improve mental and physical performance. Alpha-GPC administration increases the release of the neurotransmitter acetylcholine and facilitates learning and memory. In athletes, Alpha-GPC supplementation prevents exercise-induced reductions in choline levels, increases endurance performance and growth hormone secretion. Caffeine has been shown to increase mental focus, acuity and athletic performance, however, contributes to a nervous or anxious feeling. The purpose of this study was to measure the acute effects of Alpha-GPC supplementation in comparison to caffeine or placebo on mood, cognitive function, and physiological performance.

## Methods

Twenty participants [10 males, 10 females; 22.0 ± 3.4 years of age; height 171.9 ± 7.4 cm; weight 56.8 ± 8.6 kg] consumed 200 mg of Alpha-GPC (aGPC-L, AlphaSize^®^, Chemi Nutra, Austin, TX, USA), 400 mg of Alpha-GPC (aGPC-H), 200 mg of caffeine (CA), and a placebo (PL) in a randomized, double-blind, placebo-controlled, crossover design. Participants performed the following measurements 30 minutes after supplementation: visual analog scales (VAS) for six different moods, a serial subtraction test (SST), and tests for reaction time, hand-eye coordination, power, speed, and agility.

## Results

SST scores were 18.1% and 10.5% faster in the aGPC-L (6.19 ± 2.21 s) group compared to CA (7.32 ± 5.67 s) and PL (6.85 ± 2.52 s), respectively. Vertical Jump Peak Power was 8.5% higher in the aGPC-L (2,041.3 ± 547.2 W), 7.5% higher in the aGPC-H (2,023.1 ± 942.8 W) and 2.0% higher in the CA group (1,920.4 ± 689.6 W) in comparison to PL (1,881.9 ± 576.9 W).

The group consuming CA had significantly higher scores on the VAS for jitteriness compared to aGPC-H (p = 0.019), but not aGPC-L (p = 0.849) or PL (p = 0.086). There were no other statistically significant differences between supplement groups for any of the dependent variables.

## Conclusion

Acute supplementation with caffeine or Alpha-GPC had no statistically significant beneficial effect on measures of mood, cognitive function, or physiological performance, in part due to large individual variability between subjects. As Alpha-GPC seemed to be beneficial for certain physical and mental performance tasks, future research should focus on dosage, timing of consumption before testing measurement, bioavailability, longer term supplementation, and subject selection, in order to reduce individual variability.

**Figure 1:**
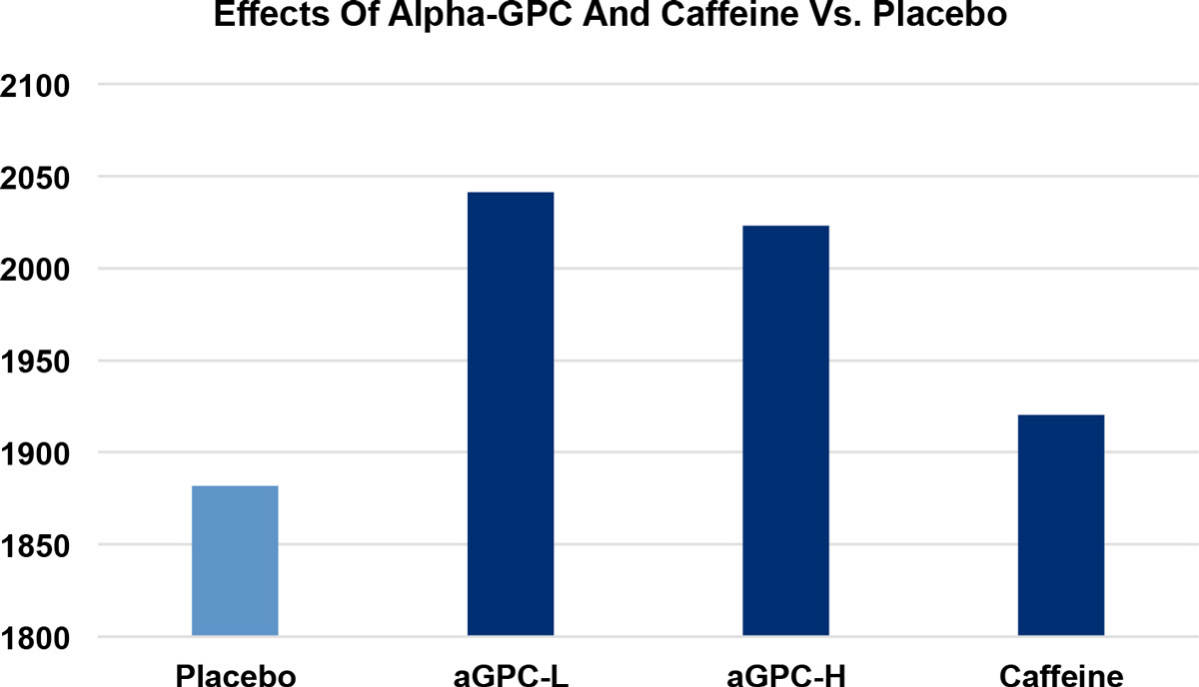
**The effects of aGPC-L (+8**.5%), aGPC-H (+7.5%), and caffeine (+2.0%), in comparison to placebo, on vertical jump peak power (in watts).

